# Supplementary material for: Targeted Sequencing of Mitochondrial Genes Reveals Signatures of Molecular Adaptation in a Nearly Panmictic Small Pelagic Fish Species
Source: Genes (Basel). 2021 Jan 13;12(1):91. doi: 10.3390/genes12010091 (PMC7828364; doi:10.3390/genes12010091)
Supplement: Supplementary file 1 [file genes-12-00091-s001.zip › Supplementary data/genes-1045925 - supplementary - tables.docx]

**List of supplementary items:**

***Supplementary Tables***

Table S1 – NCBI references sequences utilized to design mitochondrial primers

| **Species** | **Accession Number** |
| --- | --- |
| *Sardina pilchardus* | NC_009592.1 |
|  | MH329246.1 |
|  | MG729573.1-MG729589.1 |
|  | MH141161.1-MH141169.1 |
|  | AF291854.1-AF291860.1 |
|  | JQ237098.1-JQ237114.1 |
|  | FR851429.1-FR851438.1 |
|  | JQ585750.1-JQ585753.1 |
|  | JQ621900.1 |
|  | DQ197989.1 |
|  | EU224030.1 |
| *Sardinops melanostictus* | AB032554 |
| *Sardinella maderensis* | AP009143 |
| *Alosa pseudoharengus* | AP009132 |
| *Alosa alosa* | AP009131 |
| *Nematalosa japonica* | AP009142 |
| *Dorosoma petenense* | AP009136 |
| *Ethmalosa fimbriata* | AP009138 |
| *Pellonula leonensis* | AP009232.1 |
| *Clupea harengus* | KC193680.1-KC193777.1 |
| *Coregonus lavaretus* | AY778967.1-AY779006.1 |
| *Stenodus leucichthys* |  |
| *Prosopium williamsoni* |  |
| *Thymallus grubii* |  |
| *Thymallus nigrescens* |  |
| *Thymallus arcticus* |  |

Table S2 – Primers utilized to amplify mitochondrial genes.

| **Gene** | **Direction** | **Sequence (5´->3´)** |
| --- | --- | --- |
| ATP6 | Forward | ATCAATTCATAAGCCCGACCT |
|  | Reverse | GGAATAAGGGGCACGGGG |
| COI | Forward | GCGGCTTTGGAAACTGACTA |
|  | Reverse | CGGAGGCTTCATGTTGATAATTG |
| CYTB | Forward | AGCCGGAGTGATACTTCCTG |
|  | Reverse | GGGCGAGGACTAGGAAAAGT |
| ND5 | Forward | ACTCACGCTAATCTTTGCCC |
|  | Reverse | AGGCAAACTCTAGAATCGACCA |

Table S3 – Mantel test report for mitochondrial markers and microsatellite loci.

| **Marker** | **Mantel** | ***p* value** |
| --- | --- | --- |
| ATP6 | -0.18243 | 0.685 |
| COI | -0.11559 | 0.485 |
| CYTB | -0.00983 | 0.543 |
| ND5 | -0.03005 | 0.548 |
| Microsatellites | 0.38259 | 0.147 |

Table S4– Micro-checker reports for each locus, per population. In bold, loci where null alleles were detected.

| **Locus** | **Null Present** | **Oosterhout** | **Chakraborty** | **Brookfield 1** | **Brookfield 2** |
| --- | --- | --- | --- | --- | --- |
| *Bay of Biscay* | | | | | |
| SAR1.12 | no | 0.0577 | 0.0616 | 0.0551 | 0.0551 |
| **SAR1.5** | **yes** | **0.067** | **0.0738** | **0.0661** | **0.0661** |
| **SAR2.18** | **yes** | **0.3001** | **0.44** | **0.2895** | **0.2895** |
| SAR9 | no | 0.0225 | 0.0231 | 0.0219 | 0.0219 |
| SARB-A07 | no | -0.0187 | -0.0174 | -0.0169 | 0 |
| SPI7 | no | -0.0316 | -0.0256 | -0.0158 | 0 |
| *Gulf of Lions* | | | | | |
| SAR1.12 | no | 0.0337 | 0.0368 | 0.0339 | 0.0339 |
| SAR1.5 | no | 0.0396 | 0.0368 | 0.0339 | 0.0339 |
| **SAR2.18** | **yes** | **0.2513** | **0.3449** | **0.2463** | **0.2463** |
| SAR9 | no | -0.0379 | -0.0354 | -0.0345 | 0 |
| SARB-A07 | no | -0.0493 | -0.0451 | -0.0451 | 0 |
| SPI7 | no | -0.1562 | -0.0596 | -0.0267 | 0 |
| *Olhão* | | | | | |
| SAR1.12 | no | 0.0612 | 0.0602 | 0.0539 | 0.0539 |
| **SAR1.5** | **yes** | **0.0913** | **0.1004** | **0.0874** | **0.0874** |
| **SAR2.18** | **yes** | **0.1811** | **0.2201** | **0.1715** | **0.2511** |
| SAR9 | no | 0.0022 | -0.0007 | -0.0007 | 0 |
| SARB-A07 | no | 0.01 | 0.0103 | 0.0098 | 0.0098 |
| SPI7 | no | 0.0075 | -0.0063 | -0.0042 | 0 |
| *Sesimbra* | | | | | |
| **SAR1.12** | **yes** | **0.1047** | **0.1276** | **0.1065** | **0.1065** |
| SAR1.5 | no | -0.0507 | -0.0378 | -0.0366 | 0 |
| **SAR2.18** | **yes** | **0.34** | **0.5322** | **0.3238** | **0.4047** |
| SAR9 | no | 0.0603 | 0.0631 | 0.0569 | 0.0569 |
| SARB-A07 | no | 0.0526 | 0.0588 | 0.0531 | 0.0531 |
| SPI7 | no | 0.0208 | 0.0519 | 0.029 | 0.029 |
| *Tarragona* | | | | | |
| SAR1.12 | no | 0.0584 | 0.0547 | 0.0489 | 0.0489 |
| **SAR1.5** | **yes** | **0.0913** | **0.0937** | **0.0814** | **0.0814** |
| **SAR2.18** | **yes** | **0.3601** | **0.584** | **0.3449** | **0.6834** |
| SAR9 | no | 0.0372 | 0.0341 | 0.0314 | 0.0314 |
| **SARB-A07** | **yes** | **0.0674** | **0.0725** | **0.0649** | **0.0649** |
| SPI7 | no | -0.1822 | -0.0667 | -0.0335 | 0 |

**References**

Binladen, J., Gilbert, M., Bollback, J., Panitz, F., & Bendixen, C. (2007). The use of coded PCR primers enables high-throughput sequencing of multiple homolog amplification products by 454 parallel sequencing. *PLoS One, 2*, e197.

McInnes, J. C., Jarman, S. N., Lea, M.-A., Raymond, B., Deagle, B. E., Phillips, R. A., . . . Kusch, A. (2017). DNA metabarcoding as a marine conservation and management tool: A circumpolar examination of fishery discards in the diet of threatened albatrosses. *Frontiers in Marine Science, 4*, 277.
