# Supplementary figures and images for: Targeted Sequencing of Mitochondrial Genes Reveals Signatures of Molecular Adaptation in a Nearly Panmictic Small Pelagic Fish Species
Source: Genes (Basel). 2021 Jan 13;12(1):91. doi: 10.3390/genes12010091 (PMC7828364; doi:10.3390/genes12010091)

A)

### Comparing $H_o$ and $H_e$ across populations

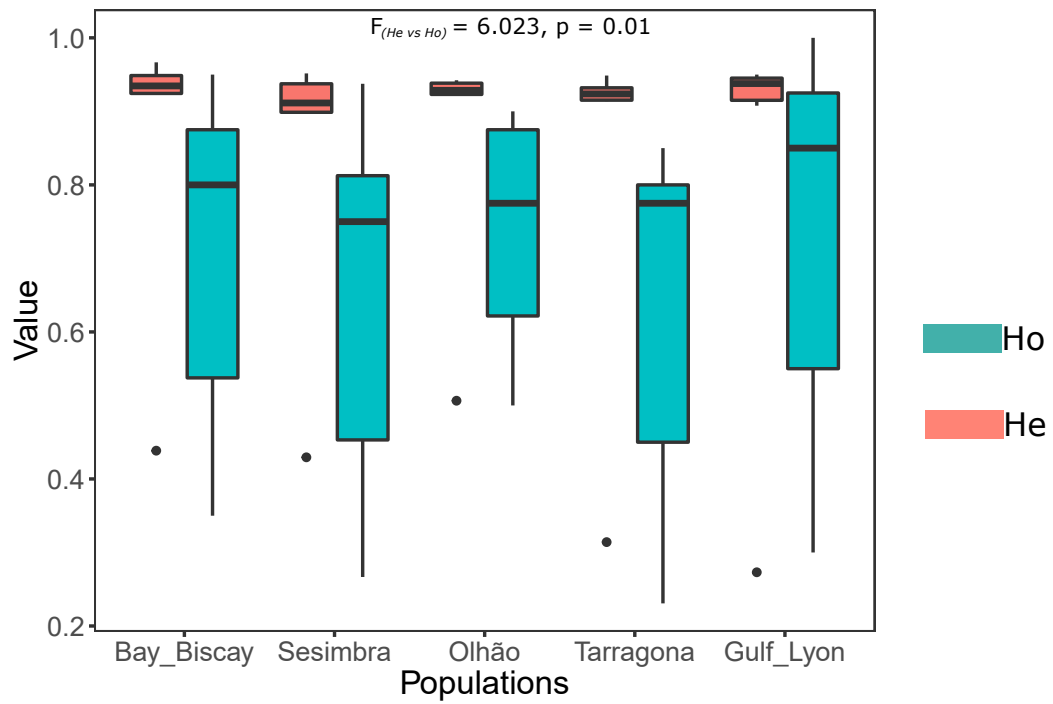

B)

### Average $F_{st}$ per locus

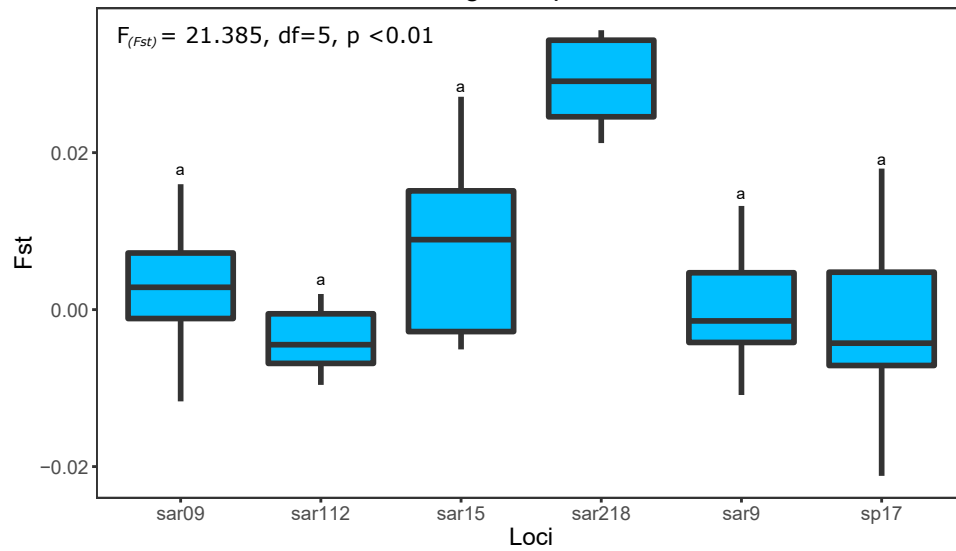

Supplement: Supplementary file 1 [file genes-12-00091-s001.zip › Supplementary data/Fig. S1.pdf]

# A)

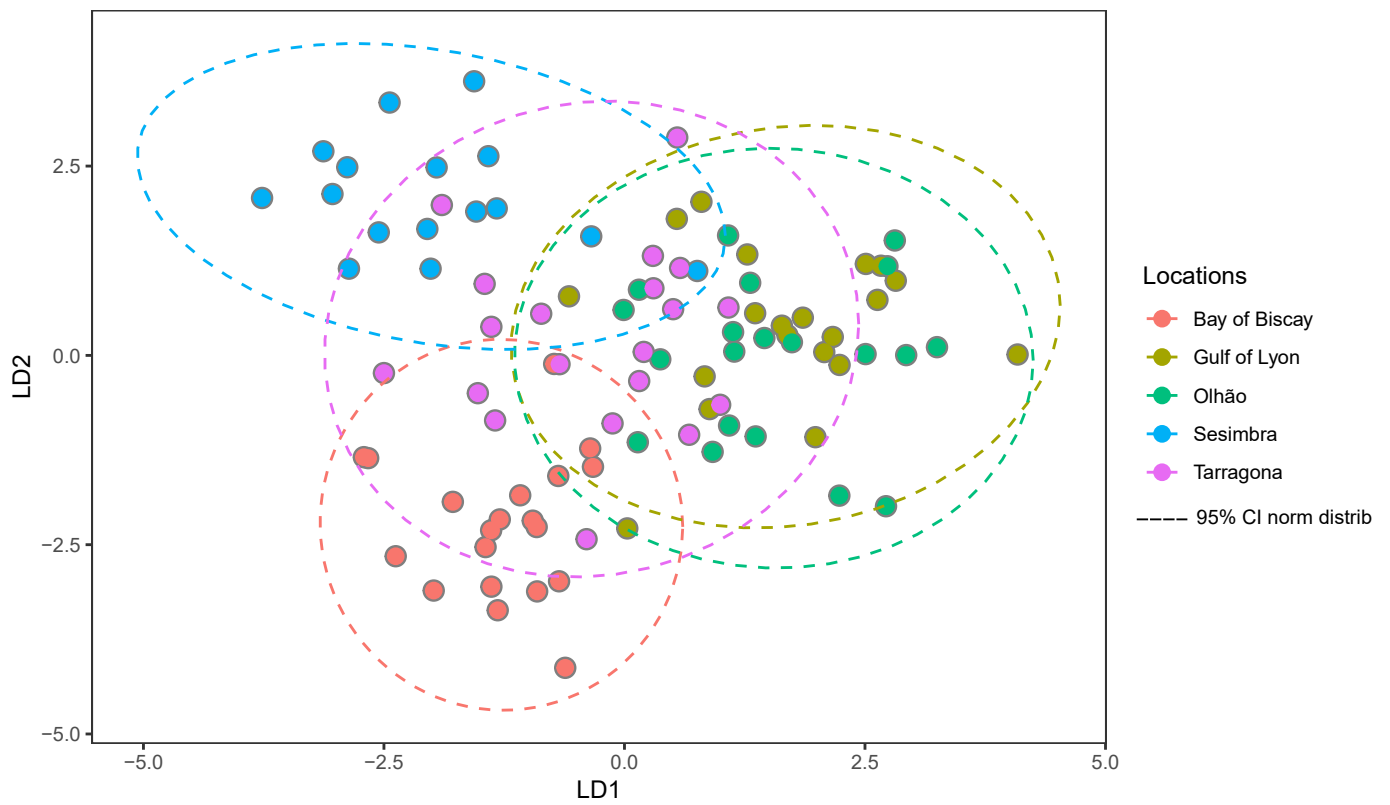

# B)

K=5

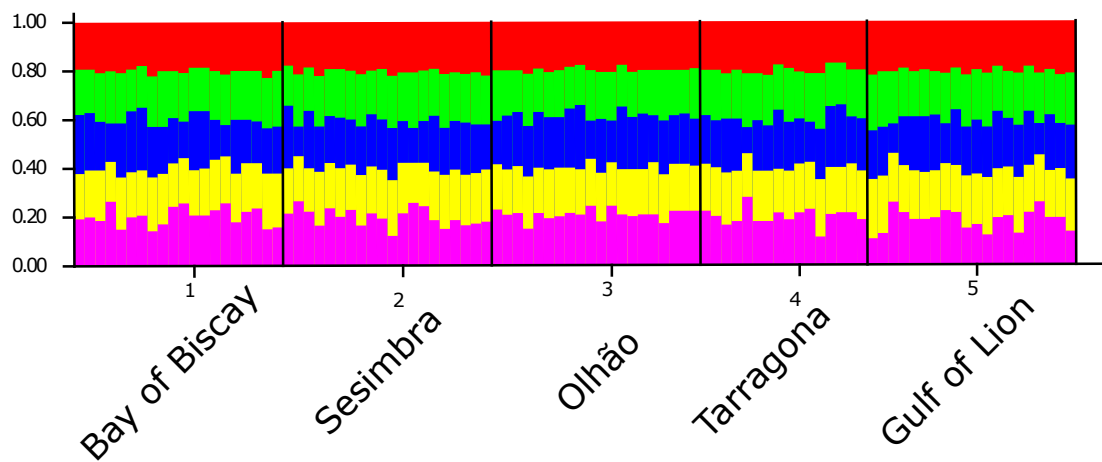

Supplement: Supplementary file 1 [file genes-12-00091-s001.zip › Supplementary data/Fig. S2.pdf]

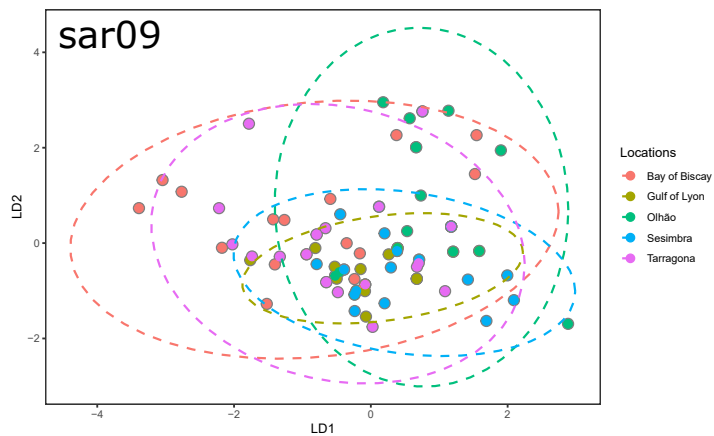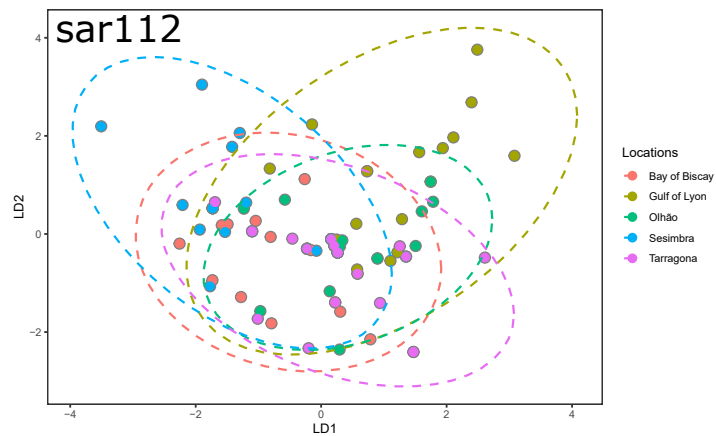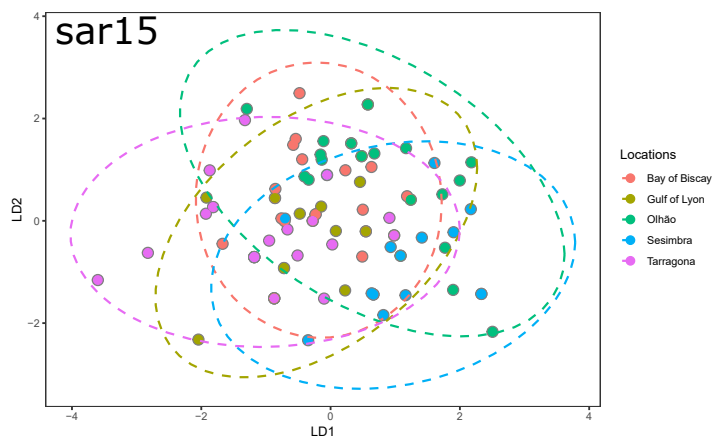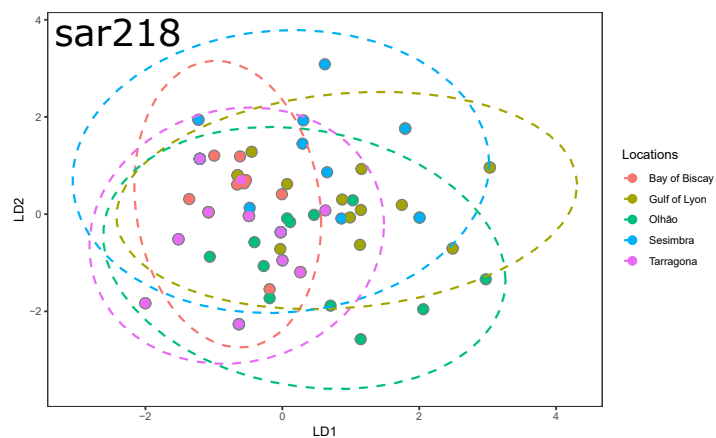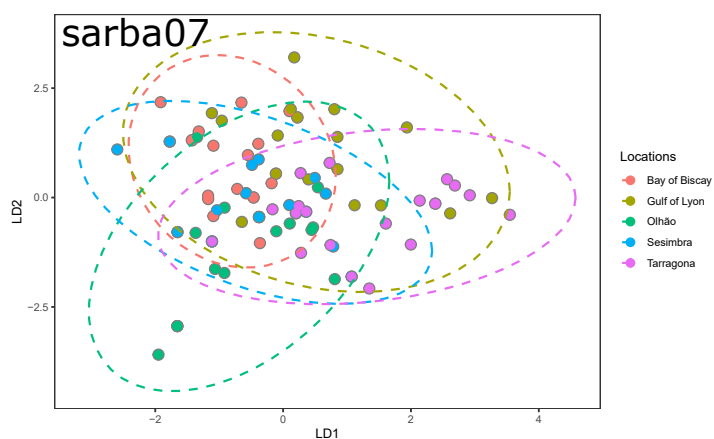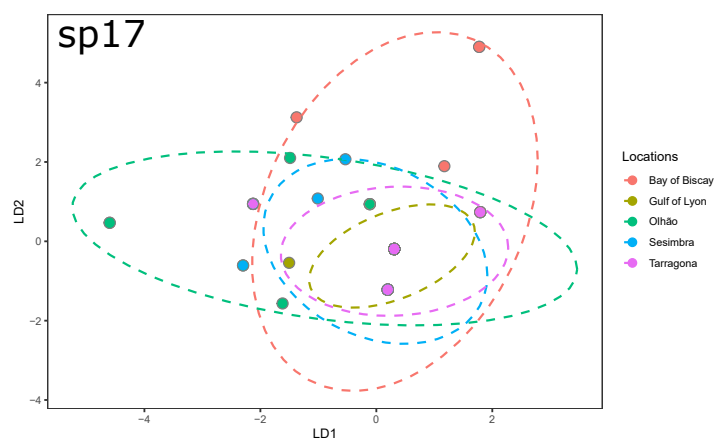

Supplement: Supplementary file 1 [file genes-12-00091-s001.zip › Supplementary data/Fig. S3.pdf]
